# An empirical evaluation of software quality assurance practices and challenges in a developing country: a comparison of Nigeria and Turkey

**DOI:** 10.1186/s40064-016-3575-5

**Published:** 2016-11-04

**Authors:** Olaperi Yeside Sowunmi, Sanjay Misra, Luis Fernandez-Sanz, Broderick Crawford, Ricardo Soto

**Affiliations:** 1 Covenant University, Ota, Nigeria; 2 Atilim University, Ankara, Turkey; 3 University of Alcala, Madrid, Spain; 4 Pontificia Universidad Católica de Valparaíso, Valparaiso, Chile

**Keywords:** Software, Software quality, Software quality challenges, Software quality assurance

## Abstract

**Background:**

The importance of quality assurance in the software development process cannot be overemphasized because its adoption results in high reliability and easy maintenance of the software system and other software products. Software quality assurance includes different activities such as quality control, quality management, quality standards, quality planning, process standardization and improvement amongst others. The aim of this work is to further investigate the software quality assurance practices of practitioners in Nigeria. While our previous work covered areas on quality planning, adherence to standardized processes and the inherent challenges, this work has been extended to include quality control, software process improvement and international quality standard organization membership. It also makes comparison based on a similar study carried out in Turkey. The goal is to generate more robust findings that can properly support decision making by the software community. The qualitative research approach, specifically, the use of questionnaire research instruments was applied to acquire data from software practitioners.

**Results:**

In addition to the previous results, it was observed that quality assurance practices are quite neglected and this can be the cause of low patronage. Moreover, software practitioners are neither aware of international standards organizations or the required process improvement techniques; as such their claimed standards are not aligned to those of accredited bodies, and are only limited to their local experience and knowledge, which makes it questionable. The comparison with Turkey also yielded similar findings, making the results typical of developing countries. The research instrument used was tested for internal consistency using the Cronbach’s alpha, and it was proved reliable.

**Conclusion:**

For the software industry in developing countries to grow strong and be a viable source of external revenue, software assurance practices have to be taken seriously because its effect is evident in the final product. Moreover, quality frameworks and tools which require minimum time and cost are highly needed in these countries.

## Background

Software application packages are usually developed under stringent conditions of time and cost while in a bid to satisfy the requirements of the users. Despite these conditions, such application or system packages must still satisfy functional and non-functional attributes such as maintainability, reliability, dependability, security, availability and other ‘ilities’ as specified. The only assurance of achieving positive results at all these fronts is by adhering to software quality assurance and management processes. To ensure that bugs and flaws in software products are identified and removed, it is necessary to adhere to software quality standards. This would prevent a number of flaws before the implementation and deployment of the application.

Software quality assurance is imperative for a software organization’s success. It ensures the quality of the software while ensuring that it is fully functional and well documented for easy maintenance. It goes beyond testing the application but also includes the monitoring and control of the entire software development processes and products (Scarpino 2011).

Software engineering is the application of a systematic, disciplined, and quantifiable approach to the development, operation, and maintenance of software, and the study of these approaches; that is, the application of engineering to software (IEEE Standard 1990). According to Sommerville (2007), it is an engineering discipline that is concerned with all aspects of software production from the early stages of system specification to maintaining the system after it has gone into use. As with other engineering fields, quality practices are necessary to attain success in the process.

Globally, this field of engineering is growing rapidly, and becoming more structured than ever. At the moment, it is more relevant than it has ever been in history. Software products are ubiquitous and changing the face of businesses globally. They are being used to monitor and deploy government infrastructures, to manage financial portfolios, carry out medical procedures, build and control real-time and mission critical systems that cannot afford to fail. Although standards in the field are not yet as pronounced and enforced as in other engineering disciplines; there are best practices and already proven quality techniques that should be taken with austerity, if quality software is to be developed.

Different software quality techniques have been developed including software testing, code reviews, process improvements, risk management, configuration and change management amongst others. These activities can be executed both manually and automatically with the aid of specialized tools.

However, this universal growth in the field is not so evident in developing countries; a negative trend is observed instead. It is indeed saddening to know that only 10% of the software products used in Nigeria are built by indigenous companies; the larger percentage comes from other countries, specifically India. It is on record that Nigeria loses an average of $1 billion dollars to software importation annually; between 1995 and 2008, N23 billion was spent on the purchase of foreign software, and in 2012 alone, over N59bn was transferred out in purchase and maintenance of software; even the government invests so much in foreign software. Nigeria has been noted to be one of the major importers of software products in sub-Saharan Africa (Nwogbo 2010; Nigerian Local Content Development Board 2012; The Ministerial Committee on ICT Policy Harmonization 2012) An explanation to all these might be that the local companies are not producing quality software products.

The quality of a software product is determined by how much the product meets the customer’s requirements, how much the product performs to specifications and the number of defects in it. It is well known, that high quality products are always patronized to the detriment of substandard ones. Therefore, a need for the assessment of the software development practices in indigenous companies in Nigeria, in a bid to unravel the cause of its low patronage and recommend possible solutions to move the industry forward is what motivated this research.

A prior research of the present work was presented in a conference (Sowunmi and Misra 2015). This work is an extension of the conference paper, including three new research questions and thus, more revealing findings. A thorough assessment of the overall software quality assurance and management of software organizations has been carried out and comparisons have been made with similar research in Turkey. The research questions investigated in totality are:

- *RQ1* Do software companies in Nigeria engage in software quality planning?
- *RQ2* Do software companies in Nigeria follow certified standardized processes and procedures?
- *RQ3* Do software companies in Nigeria engage in software quality control i.e. measure/test their software product against standards using metrics?
- *RQ4* Do software companies in Nigeria improve on their processes over time?
- *RQ5* Are software companies in Nigeria certified by international organizations?
- *RQ6* What are the challenges inhibiting the adoption of quality practices?

The instrument used to collect data was the questionnaire and the data collected was used to answer the research questions.

The next section presents a literature survey of previous works carried out in the area of software quality management and assurance, followed closely by the detailed methodology that this research work employed. The results of the findings are then be presented, followed by the discussion of results, recommendations, and conclusions.

## Background and literature survey

In this section we provide the background, concept and fundamentals of the software quality and quality assurance. The various work done in this area are also summarized in this section.

### Quality

Quality was first introduced formally by Bell Laboratories in 1916, and it gradually permeated into software production in the 1970s when military applications where being built (Lewis 2004). The term quality in the software engineering field does not apply as in other engineering disciplines such as manufacturing, in that it is not confined to predefined specifications; in this case, it should be tailored towards specific customer requirements and organizational standards (Sommerville 2007). Quality in the language of software engineering as discussed by Lewis (2004) means ‘meeting requirements’ and ‘fitness for use’. This implies that the software meets the requirements of the users as stated in the requirements specification, and it does exactly what the user needs. This definition makes the requirements engineering process and the resulting documentation very important, since the quality system revolves around it. Quality is considered a vital requirement for software products, a business essential, a competitive necessity, and a survival issue for the software industry (Murugesan 1994). It is a complex concept that is ambiguous and can be difficult to measure. Strong quality focus is emerging in all phases of the software development lifecycle with increasing emphasis on product quality, process maturity, and continual process improvements.

### Quality management

Quality management entails all planned systematic activities and processes for creating, controlling and assuring quality. It is not just a task, but it is a habit that needs to be ingrained into a company’s culture (Ebert and Dumke 2011). It also aims to monitor and refine the development process, based on the assumption that the quality of the development process directly affects the quality of the delivered product.

### Software quality assurance

There are different definitions for the term software quality assurance (SQA), some of them are stated below:

Software quality assurance, is a well-defined, repeatable process that is integrated with project management and the software development lifecycles to review internal control mechanisms and assure adherence to software standards and procedures. The objective of the process is to assure conformance to requirements, reduce risk, assess internal controls and improve quality while conforming to the stated schedule and budget constraints (Owens and Khazanchi 2009).

Software quality assurance is the planned and systematic approach to the evaluation of the quality of and adherence to software product standards, processes and procedures (Agarwal et al. 2007). It includes the process of assuring that standards and procedures are followed throughout the software lifecycle.

Software quality assurance is a process itself which envelopes the entire project and software development life cycle. It is not to be confined to the last stage of software development, or as a means of measuring the produced software. It should begin at the very onset of the project, and span through to the end or retirement of the software itself. This is because quality cannot be added to a finished product, at this stage it can only be patched; SQA is therefore a continuous process and assessment (Thayer and Fairley 1997).

It was reported in (Owens and Khazanchi 2009) that SQA consists of phases and various activities, which should be carried out by a SQA team of skilled professionals independent of the software development team. They proposed and described an SQA process framework as consisting of the following phases:

- *SQA initiation* before the commencement of a project, the SQA team is notified of it, and necessary quality control and audit processes are defined.
- *SQA planning* the goals and objectives of the software quality assurance plan are defined; quality processes or procedures to be followed, standards and metrics to be used, reviews and audits to be carried out are decided.
- *Requirements assurance* validation of requirements to ensure testability, feasibility and completeness.
- *Design assurance* verification of design against requirements, and ensuring that the planned methodologies are being used.
- *Development assurance* making certain that the development team is following the stated development process and coding standards.
- *Testing assurance* verifying that adequate testing has been carried out and defects nave been tracked, recorded and corrected.
- *Implementation assurance* providing assurance that the necessary implementation steps have been completed prior to and after implementation.

*SQA closing* this entails confirming that the necessary project closing activities, post project review and formal documentation of lessons learnt have been completed.

The term software quality assurance is generally used interchangeably with software quality management, likewise in this work.

### Quality planning

This is the process where a specific quality plan is developed for particular project. It involves a selection of organizational standards that are specific to the software project in question and the development process to be used. It also specifies how the quality assessment process will be carried out. It helps to evaluate the project at its end, by checking whether the plan and all quality milestones are achieved.

### Quality control

This is the process of monitoring the software development process and checking the product or deliverables (such as the design model or code) to make sure the quality plan and organizational standards and procedures are being followed by the development team. Quality control encompasses a set of software engineering actions that help to ensure that each work product meets its quality goals (Pressman 2010). It can be carried out using automated software assessment or by a quality review team. It often involves measurements using software metrics. Any compromise to quality standards that is detected is documented and forwarded to the appropriate personnel for correction. Methods that can be used include design and code walkthroughs, review, testing, inspection and performance checks.

### The software quality assurance team

Every member of the overall project team is responsible for maintaining quality in the project, not withstanding, there is still a dire need for a dedicated team committed to the purpose of quality assurance. In previous years, quality assurance was the responsibility of whoever built the product, but that is not so anymore. This team should comprise of people separate from the development team. They assess the product from the customer’s point of view. Their responsibilities include testing, review of documentation (development plans, testing plans, project plan) for completeness and adherence to standards, periodic inspections, reviews and audits (Godbole 2004).

### Costs and benefits of software quality assurance

The need for software quality assurance cannot be overemphasized. A lack of it has been shown to be one of the major causes of software project failure. It plays a very vital role in the software life cycle process and can substantially increase the chance of a project’s success. It also helps to mitigate potential risks (Owens and Khazanchi 2009).

Regardless of the tools, techniques and experience of the development team, failure to give heed to software quality can result in exceeding the allocated time and budget for the project, failure to meet project objectives, poor customer satisfaction and excessive rework.

Software quality is not achieved by chance; a product does not just attain the specified requirements by sheer luck. It is the result of deliberate actions and steps which cost time, money and effort. While ensuring quality has a cost, lack of quality has a cost too. The cost of quality can be divided into three: cost of prevention, cost of appraisal and cost of failure. Costs of prevention include costs to plan and coordinate activities in the SQA process; appraisal costs include cost of measuring the product such as testing, review and metrics evaluations while cost of failure include cost to correct an error, or rework a process due to defect. Failure costs can be internal based on defects detected before shipment to the client or external, based on defects detected have deploying at the client’s site (Pressman 2010).

In the long run, quality management decreases production costs because the sooner a defect is located and corrected, the less costly it will be. While the initial costs can be very substantial, it cannot be compared to the adverse effects of losing a customer, a bad reputation, or going out of business. The costs of prevention are easier to bear, than the cost of failure (Lewis 2004).

### Challenges inhibiting implementation of software quality assurance

Software companies frequently face many difficult challenges in their attempt to deliver high-quality software and strife to achieve customer satisfaction (Elgebeely 2013). From different literatures, possible factors that can impair software quality management include: impatient management, strict deadlines, developer ego, extra cost required (e.g. for the purchase of tools), bureaucracy, inadequate tools that can help to automate the process, low level of acquaintance and knowledge of the process, lack of organizational training on quality standards, inexistent framework for quality management in the organization, disapproval by top management, contrary beliefs and opinion, and previous futility of the process.

### Pitfalls in SQA

From the literature review, a number of general pitfalls practiced by software organizations in a an attempt to ensure quality were identified and discussed in this sub-section.

Software organizations tend to rush into implementing a software quality assurance process without a prior establishment of functional software quality assurance practices within individual departments (Scarpino 2011). Ideally, the reverse is supposed to be the case, quality assurance needs to be enforced first at the departmental level before an encompassing overall process at the top level.

Some software organizations avoid enforcing quality assurance processes in an attempt to ‘cut cost’ and ‘save time’. This is wrong because research has shown that bugs are cheaper to identify and correct during development than after release or deployment at the client’s site (Drake 1996).

Software organizations need to observe and improve their SQA processes from time to time. When an established SQA process or activity is being applied for different projects, the suitability and effectiveness of the process should be monitored for future improvements. However, due to some factors this is not usually implemented and improvements are not made.

Evading some already established processes and/or not adhering strictly to the specified order. Each stage or activity in a SQA process is necessary and essential for the overall effectiveness of the entire process. The results of the overall process cannot be relied upon if the sequence of steps laid down is not duly followed.

Mix-up of roles is another issue. A number of organization mixup roles of personnel in executing some tasks. For example, a development manager closing bugs in the bugs repository after they have been fixed rather than a QA team member, members of the development team managing the requirements document, a developer who also serves as a support staff. All these might make void the essence of the process.

SQA should not be seen as the sole responsibility of the SQA team, but a responsibility of everyone involved in any activity in the entire software development lifecycle. Every worker should be thoroughly informed of what is expected in ensuring quality in whatever role they take part in. Moreover, SQA is much more than testing and should not be delayed until the latter end of the project, rather it should be incorporated right from its inception.

### Related works

Generally, quality management processes are not strictly adhered to by software companies, and this reduces the overall quality of the software produced. Several research have been carried out with respect to quality implementations in the development processes of software organizations.

Drake (1996) presented a case study that showed the benefits of ‘applied quality assurance and code-level measurement activities’. The case study presented a software package that had a time-line of 6 months for development, integration and delivery. Due to the tight schedule, throughout the development period, QA activities such as code inspections, walkthroughs, process control and testing were neglected. At the end of the project, the users considered it unacceptable because it took about 4–5 h to perform its critical function. After 2 weeks of an attempt to fix the code, the senior developer realized that the code needed to be reengineered. After about 6 weeks, the new code was ready and that critical section took only few seconds. Due to lack of enforcement of quality, more time and effort was eventually spent.

Laporte et al. (2012), reported the results of a research that measured the cost of software quality. The results from analyzing over 1100 software tasks that spanned about 88,000 h showed that software quality accounts for about 33% of overall project cost—cost of evaluation accounting for the highest (21%), cost of correcting anomalies was next with 10% and then cost of the prevention, the least, at 2%. It cannot be overemphasized that it pays off to carryout preventive measures of ensuring software quality rather than corrective measures.

Researchers have also worked on the impact of organizational factors on quality. Nagappan et al. (2008), carried out a research to provide empirical evidence to validate that organizational factors affect software quality. The authors developed a metric for measurement and applied it to data from Windows Vista. Their results showed that of a truth organizational factors affect failure-proneness, even above metrics like churn, dependencies, complexity. Lavallée and Robillard (2015) also carried out a study to determine how organizational factors affect working conditions of software developers and in turn the quality of software produced. It was observed that decisions made under pressure due to certain organizational factors such as structure of the organization had a negative effect on software quality. The study was carried out via non-participant observation during weekly meetings of an in-house development team of a large telecommunication company over a period of 10 months. Organizational factors including budget protection, scope protection, organizational politics, human resource planning issues and undue pressure from management and senior developers negatively affected the quality of the software products.

Even for companies which implement SQA practices, different issues impede the success and full realization of the benefits of the process. Scarpino (2011), conducted a software quality assurance evaluation on a software organization that develops software for mobile data synchronization and manages software systems. The research which focused on a particular organization was conducted via face to face interviews at the organization. The findings from the research revealed that the organization was more into software testing rather than an entire software quality process. The research revealed a number of issues within the organization: the organization’s test case steps were too bulky, the test case layout was not directly related to functional specifications, e-communication was employed instead of physical communication between members of the QA team and the developers to analyze test activity, lack of involvement of the QA group at the initiation of a change, lack of efficient use of test case and defect repositories (they were not being used as knowledge bases with other relevant departments; the bug tracking tool (Bugzilla) and the test case repository were not being used as expected) mixup of roles between the development manager and the QA team, as well as insufficient communication between the technical, QA and development team.

Scarpino and Kovacs (2008) also researched on the adverse effects of implementing a SQA tool without prior establishment of a software quality process for the organization. An organization that implemented an SQA tool was used for this study. The data was collected via interviews and open observational analysis by an external consultant and an internal QA expert. The following were the findings: team members to use the tool were not given adequate training and assistance, there was no clear documentation of how the system would fit into the company’s software development life cycle, the short time and a lack of initial communication with members of the team led to high resistance towards the implementation of the tool. The tool itself was not properly reviewed to verify that it offered all the company’s expectations. The researchers also noticed an inconsistent review of the implementation progress of the tool.

More specifically, assessment of software quality practices of organizations have also been carried out. An empirical study was carried out in (Pusatli and Misra 2011a) to evaluate the proper implementation of measurement and metric programs in software companies in an area in Turkey. From their research, they observed a common reluctance and lack of interest in utilizing measurements/metrics despite the fact that they are well known in the industry. They also discovered that internationally recognized standards such as ISO and CMMI are only followed if they are explicitly specified as a project’s requirements.

An assessment of the implementations of quality standards in the software industry of Turkey was also carried out (Pusatli and Misra 2011b). They found out that even organizations that have the ISO and CMMI certificates do not follow the prescribed directives of this organization after obtaining the certificates. They found out the companies do not see quality issues as primary, some don’t even know the names of common quality standards; they believe acquiring the standards are just for ‘show-off’ and that they do not necessarily influence the quality of the products, neither do they make the customers happy which is their priority.

Within the context of developing countries, specifically in Nigeria, similar work has also been done.

Soriyan and Heeks (2004) performed a comprehensive study of the Nigerian software industry. Their study cut across a general profile of the industry, reviewing location and ownership of the firms, their personal and job descriptions. The study also covered the type of customers they provide services for, as well as the products and services rendered, not leaving out the processes and methods engaged in executing projects. As a result, an expansive picture of the general state of the software industry in Nigeria at the time of the study was presented. However, the study only gave a general profile on the industry without focus or emphasis on its SQA practices.

A group of researchers also investigated the state of software engineering ethics in Nigeria. They observed nonchalance, dispassion and mass negligence on the issue. They also showed with the aid of a case study, that the ACM/IEEE software engineering code of ethics when applied to software development project helps to resolve ethical dilemmas (Ume and Chukwurah 2012).

A research to feel the pulse of software professionals in Nigeria on their perceptions of the software inspection as a software quality assurance activity was carried out in (Akinola et al. 2009). The authors used a structured questionnaire research instrument for their work. They found out that software inspection is highly neglected in most organization’s software development process, as they consider it a waste of time.

Olalekan (2005) reported a discourse on the state of the software industry in Nigeria. The research highlighted ‘process compromise’, ‘resistance to measurement’ and poor training of students at the higher education institutions as some of the problems befalling the industry. However, the authors only adduced reasons for its mature state, no empirical investigation was carried out.

More closely related is the work by (Aregbesola et al. 2011) who carried out an assessment of how and to what extent software organizations in Nigeria follow organizational processes. Their survey revealed that the companies do not have proper documentation of their organizational software processes and they only apply implicit in-house methods. Using the Software Engineering Institute (SEI) CMMI, model and the SEI Maturity Questionnaire, they measured requirement management, software project planning, software project tracking and oversight, software subcontract management, SQA, and software configuration management. Based on the software process maturity assessment and capability assessment of the industry, the Nigerian software industry is only at the SEI CMMI maturity level 1, while it toggled between 0 and 1 in key process areas.

All these works individually assessed only a part of the entire software quality management process. This research on the other hand takes another dimension, as it seeks to assess the entire processes involved in software quality management and not just a part of it. It also goes beyond that to identify the challenges inhibiting the practice of software quality which the reviewed research works did not assess, this is to discover the peculiarities in the environment that contribute to the current state, so that suitable solutions can be proffered. Moreover, a comparison with the state of the industry in Turkey is made based on the report from a previous research.

## Research methodology

The quantitative research method was applied in this research. The survey technique was used and the qualitative data obtained was analysed using descriptive statistics. A thorough literature review of the activities involved in software quality assurance management was embarked to develop the research questions and the research instrument, a closed-ended questionnaire. The questions were reviewed, validated and verified by a software quality professional and a statistician to ascertain the suitability of the questions. A pilot survey was then conducted to ensure that respondents have the correct understanding of the questions.

The questionnaires were then distributed to stakeholders in software development in Lagos being the hub of software activities in Nigeria, and the home to nearly 50% of all software firms in Nigeria (Soriyan and Heeks 2004). The data collected was collated and analyzed.

**Fig. 1:**
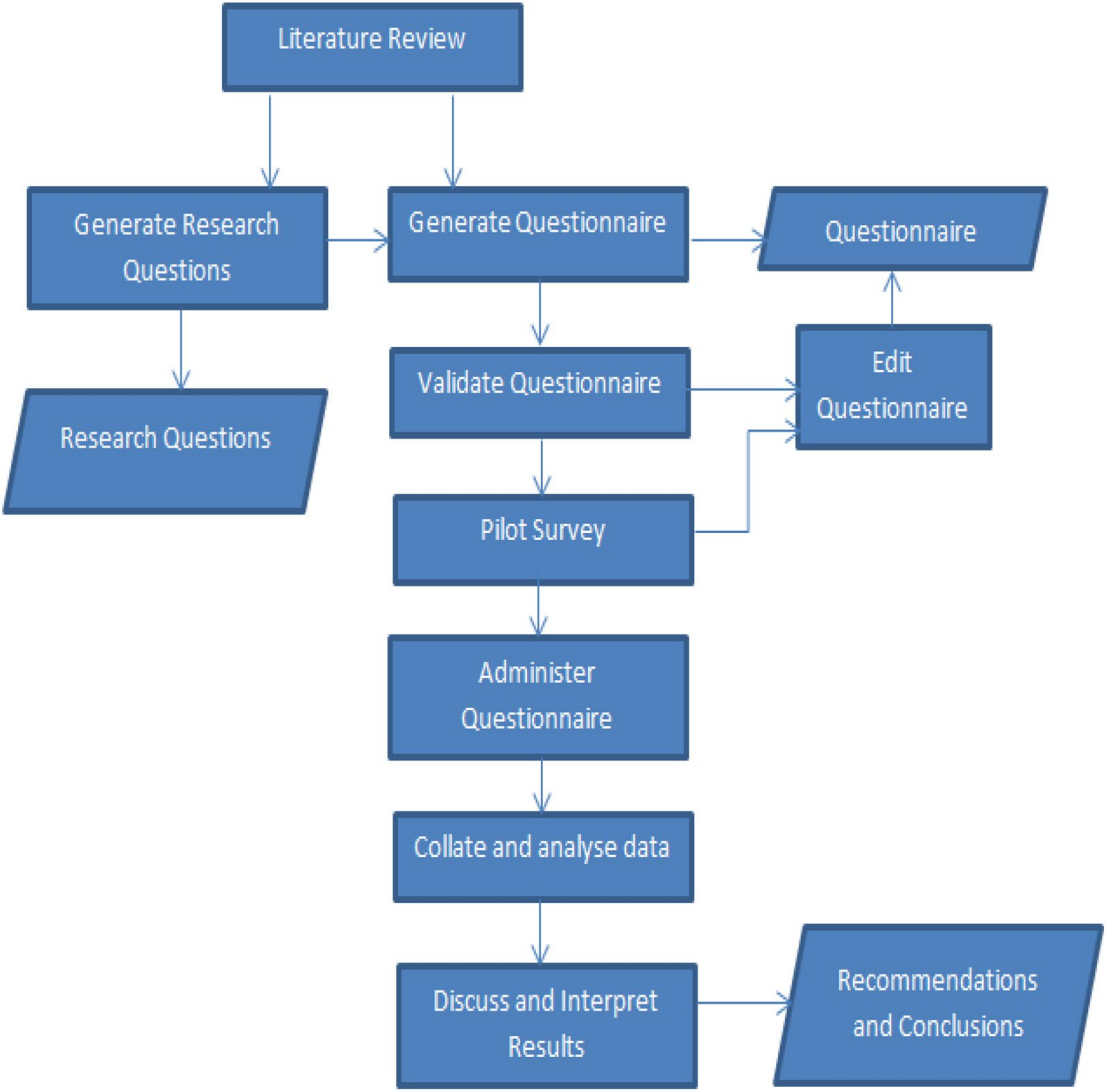
Research methodology

Furthermore, the internal validity for different sections of the questionnaire was measured using the Cronbach’s alpha. This coefficient was calculated using IBM’s SPSS (Statistical Package for the Social Sciences). The results are discussed, and based on the findings, conclusions made. Figure 1 illustrates the research methodology.

## Results

This section details the full results of the entire work. The results of the additional research questions are included. A total of 86 questionnaires were analysed. To estimate the reliability of the research instrument, its internal consistency was measured using standardized Cronbach’s alpha which is also known as the coefficient alpha. This was calculated on different sections of the questionnaire, because they measured separate entities of the SQA and also had different Likert scales. For the section that measures quality control and standards, the cronbach alpha was 0.734, for the section that measured quality planning, the cronbach alphas was 0.689 while it was 0.809 for the section that measured the challenges. In the interpretation of cronbach alpha, 0.00 means no consistency, 1.0 means perfect consistency, and any value from 0.70 implies acceptable consistency, as such we can conclude that the research instrument is internally consistent, therefore reliable.

**Fig. 2:**
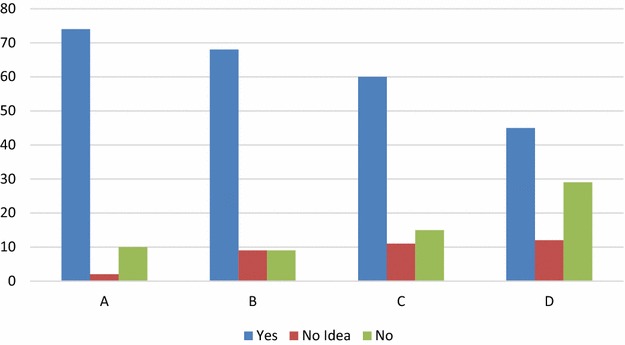
Quality standards

The analysis of the data gathered is as follows:

On quality standards Table 1 and Fig. 2 report the findings. 11.6% of the respondents reported that their organizations did not observe quality standards while only 2.3% said they have no idea of what quality standards are.

**Table 1:** Quality standards

Questions	Yes	No idea	No
Do you have defined organizational processes for software development in your company? (A)	74	2	10
Are members of staff usually trained on the quality standards of your organization? (C)	68	9	9
Does your organization have a software quality assurance team? (D)	60	11	15
Is your organization’s quality assurance team different from the project development team? (E)	45	12	29

Only 33.7% do not have a SQA team that is separate from the development team, and 30.1% either do not have a SQA team or know about such a team.

**Fig. 3:**
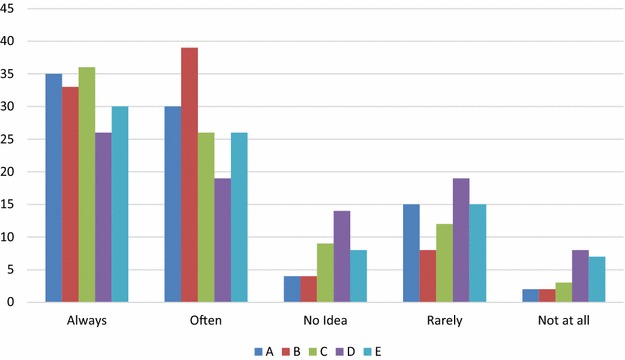
Quality planning

Results on quality planning are reported in Table 2 and Fig. 3. A total 22.1% respondents reported that they rarely or never carry out quality planning activities, while only 36 respondents of the 86 reported that they always carry out risk management activities.

**Table 2:** Quality planning

Questions	Always	Often	No idea	Rarely	Not at all
Do you develop a quality plan for new software projects before their commencement? (A)	35	30	4	15	2
How often do you use a project schedule plan for each software project? (B)	33	39	4	8	2
Do you carry out risk management activities for your software projects? (D)	36	26	9	12	3
Do you use a budget plan for every software project? (E)	26	19	14	19	8
Do you plan the tests and reviews (e.g. write the test cases) in advance	30	26	8	15	7

**Fig. 4:**
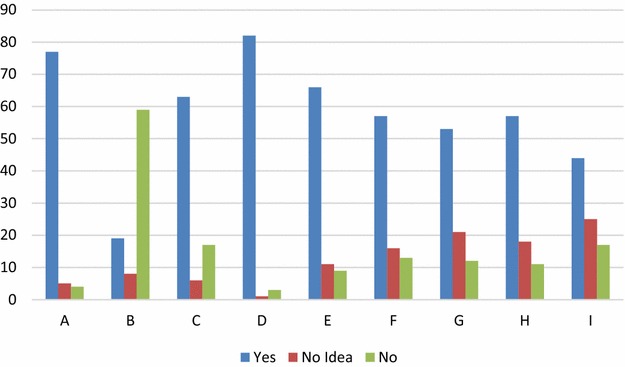
Quality control and measurement

As seen in Table 3 and Fig. 4, quality control and measurement activities are carried out, but only 22% reported that they employ an external review team on their projects. However, periodic reviews, software testing and code walkthroughs are judiciously carried out.

**Table 3:** Quality control/measurement

Questions	Yes	No idea	No
Do you carryout periodic reviews on on-going software projects? (A)	77	5	4
Do you employ an external review team for your software projects? (B)	19	8	59
Do you carryout review of your software documentations? (C)	63	6	17
Do you carry out testing of software before releasing it to customers? (D)	82	1	3
Do you carryout regression testing of software after making any modifications? (E)	66	11	9
Do you engage in code walkthroughs as a means of inspecting codes? (F)	57	16	13
Do you engage in design walkthroughs before coding? (G)	53	21	12
Do you carry out software quality assurance audits? (H)	57	18	11
Do you have a configuration management and change control system? (I)	44	25	17

On process improvement activities, 75.6% reported that they improve their processes based on metrics from the previous project, however, this has not been certified by any organization. From the first round of the survey as 57% do not even have an idea of the CMMI, and only 16% are registered under the ISO 9000 assurance models. From the second round of the survey, from the additional questions included, 86.3% are not aware of international or national software standards, and as such are not planning to adopt any.

**Fig. 5:**
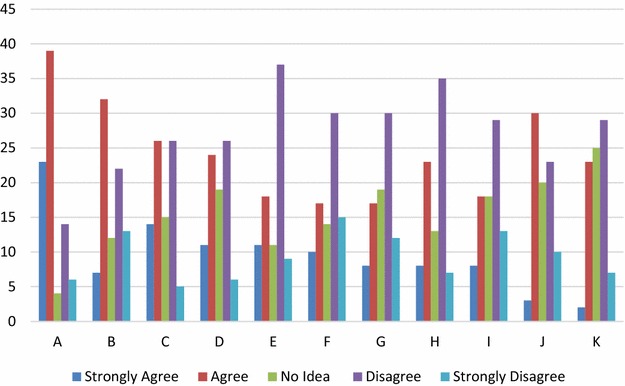
Challenges

From the data gathered from respondents, one can ascertain that challenges are being faced at attempts to adhere to software quality assurance practices. Out of the 10 challenges highlighted, the most prominent ones identified include: strict deadlines 72%, extra cost required 46%, inadequate manpower 45.3%, and bureaucracy of the process 40.7%. Full details are given in Table 4 and Fig. 5.

**Table 4:** Challenges

Questions	Strongly agree	Agree	No idea	Disagree	Strongly disagree
Strict deadlines (A)	23	39	4	14	6
Developer ego (B)	7	32	12	22	13
Extra cost required (e.g. for the purchase of tools) (C)	14	26	15	26	5
Bureaucracy involved in the process (D)	11	24	19	26	6
Inadequate automated tools (E)	11	18	11	37	9
Low level of acquaintance and knowledge (F)	10	17	14	30	15
Inexistent framework for quality management in the organization (G)	8	17	19	30	12
Lack of adequate training about the organization’s quality framework (H)	8	23	13	35	7
Disapproval by top management (I)	8	18	18	29	13
Contrary beliefs and opinion (J)	3	30	20	23	10
Previous futility of the process (K)	2	23	25	29	7

## Discussion

Sixty-nine of the eighty-six respondents i.e. 80.2% were male while only 19.8% (17) were female. The organizations were of varying staff strength but mostly between 5 and 15.

From the results, some of the striking findings include the following: 13.9% of the respondents either do not have any idea of, or do not practice software quality standards.

Quality standards being major ingredients of quality software is still not understood even in the smallest measure by some practitioners. This implies that in their software development projects, quality standards are not maintained or considered at all.

33.7% of respondents do not have a separate SQA team. As important as a SQA team is in a software development organization, more than half of the respondents do not have one. This implies that no form of quality check is made on software packages before they are shipped to the customers except those made by the developers. This is very risky as it usually takes another eye to identify a bug or potential risk in a software application. 35% do not even have a SQA team at all, or do not have an idea of what a SQA team is.

59.3% do not carry out quality planning always. This means that at the onset of software projects, the quality expectations of the software products are not clearly spelt out. This makes it difficult to determine at the end of the day if the quality attained is what was expected.

Risk management activities in software quality assurance has less than 30% awareness on the part of practitioners of software, this is not a positive one, because it shows that potential risks are not taken care of ahead of time. It they eventually occur; they can really destabilize the team or even crash the project.

81% do not carry out external reviews; that is, they do not subject their software development to scrutiny by parties that are not a part of the organization.

Though some aspects of software quality assurance are taken care of, there is no evidence that certified standardized processes and procedures, are followed.

A good percentage attested to the fact that they adhere to quality standards and control, however, a considerable number are yet to align to this, as such need to be sensitized. Adequate reviews are not being carried out due to the absence of a separate SQA team and an external review team by most organizations. It is not efficient to have those who worked on a project to also review it. A majority of the respondents were not aware of the CMMI as 57% said they had no idea about it at all, and only a very few are registered to the ISO 9000 quality assurance model or any software quality standard organization.

While the practitioners claim to be following software standards, these standards are only based on their level of their knowledge and not aligned to industry standards, as such they might not be yield the best of results.

For the result on challenges, top on the list of barriers was strict deadlines which means that when the time to market is very close, a lot of steps to ensure standards are bypassed. Contrary beliefs and opinion, developer ego, bureaucracy involved in the process and the extra cost involved are other major inhibiting factors. From the first round of the survey we also find that inadequate planning and manpower are also inhibitors.

A majority of the software developers that work in this organizations partake in a minimum of 3 phases of the software lifecycle, this shows that the same set of people are involved in different aspects of a project simultaneously which is not a very good practice because, a likely error committed might not be discovered.

### Comparison

This section presents the comparison with a similar study conducted in Turkey (Pusatli and Misra 2011a) which was conducted to determine the level of adherence of small and medium scale software enterprises to quality standards.

With respect to compliance with international standards organizations, similar results were obtained. Our study showed that more than 50% of software practitioners are not aware of these standards. The research in Turkey indicates greater awareness of these standards but they are only pursued when they are explicitly required for a project or a job at hand, otherwise, they are seen as long-term goals. Reasons for not taking up the CMMI certification given by some respondents include that it slows the development process and it is not so efficient in practice for small software companies. However, it was observed by the researchers that some software companies that attain the certification only have it as a label and do not follow the regulations afterwards.

Just in line with one of the major challenging inhibitors observed in Nigeria “Strict deadlines”, the review in Turkey identified the same challenge and revealed that the main aim of practitioners is usually to complete and deliver a project within the tight timeline given.

The research in Turkey found that the academic background of the practitioners also limited their knowledge on quality standards, this is because courses on software quality taught in the universities are electives and not compulsory, as such not all graduates of software engineering are grounded in the area.

*Other general similarities are discussed* quality requirement is not seen as priority, some companies are not aware of quality standards and tools that exist to enhance the measurement of quality. Financial constraints hinder quality, e.g. the cost of hiring extra hands to constitute the SQA team or a professional SQA expert.

This study was conducted in the South Western part of Nigeria only, specifically Lagos, because it has been identified as the hub of the industry in Nigeria, however, this research can be extended to other parts of the country. Moreover, the comparison made with Turkey was based on a previous research, and no new empirical investigation was carried out.

## Recommendation

Having discussed the results and findings, the following are recommended.

The Institute of Software Practitioners of Nigeria (ISPON) should sensitize its members on the importance of adherence to quality standards and practices, because a number of firms see it as an extra process with extra cost attached and no remuneration. They should be informed that while enforcing quality might seem expensive at the onset, it is actually cheaper, because not conforming might be costlier in the long run.

Furthermore ISPON can establish a set of quality standards to act as a guide nationally. These standards should be adopted from existing internationally acclaimed standards but made suit the peculiarities of the Nigerian software industry. The institute should not just formulate the standards, but ensure that software practitioners adhere strictly to them.

Software practitioners in Nigeria should also be informed of international institutes and standards organizations that exist to govern and accredit software practices, because it was observed that a vast majority do not even know these organizations as important as they are. They should not just be informed, but also thrive to get accredited by them. This will set the industry in the global stage and make them fit for large and international projects, because they serve as requirements for most of them.

Software companies should of necessity set up a SQA team which ideally should be separate from the development team. They should not partake in any other phase of development, so that they can be properly positioned to identify flaws in the software and other products. Members of the development team should be adequately trained not only on the technical aspects, but also on the quality standards of the organization, and regulatory bodies in the industry both nationally and internationally.

Furthermore, automatic static analysis (ASA) can be employed by these firms since they have limited resources, (in terms of finance, manpower and experienced personnel), and need to make efficient use of these resources.

Automatic static analysis has been proven to be effective, capable of detecting major flaws in program codes, while requiring little effort. They can be incorporated into their existing QA processes, to make it stronger and more reliable. It will help to save the time expended on manual code walkthroughs, and uncover errors usually overlooked mistakenly in the manual process. It will also save cost because some open source packages are actually available for use e.g. ConQAT.

A good awareness of the difference in the cost of ensuring quality during development, before delivery as against after delivery to clients will let organizations see that they can save a lot of time and stress by ensuring quality, because the general notion is that it is not so necessary.

Institute of Software Practitioners of Nigeria and individual organizations should also organize or sponsor their members or employees to attend non-vendor specific conferences and also research in new ways and tools that can help to improve quality efficiently.

Organizational factors that affect productivity such as culture and structure should be properly reviewed and re-defined where necessary, to enhance adherence to software quality processes, because research has shown that they are related and should not be neglected.

Software quality assurance tools can be implemented to reduce the time and effort of team members on quality assurance; however, they should only be implemented after verifying that the tool suits the organization’s SQA process and there would be adequate training of personnel to use it.

Research in the area of software quality should also be sponsored and higher institutions should make software quality assurance a major/compulsory course for students specializing in software engineering.

## Conclusion

The research has assessed the overall software quality assurance practices of practitioners in a developing country. The research which was spurred by the need to reduce the level of importation of software into Nigeria and increase the level of patronage of indigenous software organizations has unveiled some potential reasons for the current state of the industry. Recommendations have been made to tackle the current menace and improve quality software practices which if adhered to would lead to the production of quality software packages that would be patronized and stand the test of time.
